# Ultrasound-Derived Stretch Reflex Threshold Estimation Using Tendon-to-Bone Distance During Tendon Tapping in Post-Stroke Spasticity

**DOI:** 10.1109/TNSRE.2026.3705277

**Published:** 2026

**Authors:** Seongyeon Yang, Matthieu K. Chardon, Vaheh Nazari, Zhen Song, Yongping Zheng, Sungjin Bae, William Z. Rymer

**Affiliations:** Shirley Ryan AbilityLab, Chicago, IL 60611 USA; Department of Neuroscience, Feinberg School of Medicine, Northwestern University, Chicago, IL 60611 USA, and also with NAISE, Argonne National Laboratory, Lemont, IL 60439 USA; Department of Biomedical Engineering, The Hong Kong Polytechnic University, Hong Kong, SAR, China; Department of Biomedical Engineering, The Hong Kong Polytechnic University, Hong Kong, SAR, China; Department of Biomedical Engineering and the Research Institute for Smart Ageing, The Hong Kong Polytechnic University, Hong Kong, SAR, China; Shirley Ryan AbilityLab, Chicago, IL 60611 USA, and also with the Department of Physical Medicine and Rehabilitation, Feinberg School of Medicine, Northwestern University, Chicago, IL 60611 USA; Shirley Ryan AbilityLab, Chicago, IL 60611 USA, and also with the Department of Physical Medicine and Rehabilitation, Feinberg School of Medicine, Northwestern University, Chicago, IL 60611 USA

**Keywords:** Deep tendon reflex, spasticity, stretch reflex threshold (SRT), stroke, ultrasound imaging

## Abstract

Spasticity assessment after stroke requires objective measures of stretch reflex excitability, yet clinically practical and structurally interpretable approaches for identifying stretch reflex thresholds (SRTs) remain limited. We present an ultrasound-derived method for quantifying SRTs during controlled tendon tapping by tracking tendon-to-bone (T2B) displacement, which reflects local musculotendon deformation. Ten individuals with chronic stroke participated. A LinMot tapper delivered graded mechanical taps to the distal biceps tendon. A B-mode ultrasound probe integrated into the tapper tip tracked T2B displacement, while a force sensor and surface electromyograms (EMG) recorded reflex responses. T2B reflex amplitude was defined as the post-tap peak tendon displacement relative to its pre-tap position. As indentation depth increased, T2B reflex amplitude exhibited a nonlinear-to-linear pattern, enabling clear identification of SRT as the transition point between the two. T2B reflex amplitude showed strong within-subject correlations with reflex force and rectified integrated EMG (RIEMG) (force: mean *r* = 0.86; RIEMG: mean *r* = 0.82), comparable to the force–RIEMG correlation. T2B-based SRT estimates demonstrated within-session repeatability (CV = 10.48 ± 10.06%), comparable to forcebased SRTs and lower than RIEMG-based SRTs. Baseline T2B preload distance varied across individuals and was positively associated with SRT, suggesting that a structure-informed interpretation may provide physiological context beyond externally imposed indentation depth.

## I. Introduction

STROKE is a leading cause of long-term disability, often resulting in persistent motor dysfunction [1]. Many stroke survivors develop spasticity, a sensorimotor disorder characterized by stretch reflex hyperexcitability [2], [3]. Even small changes in muscle length can trigger exaggerated reflex responses that contribute to increased joint stiffness and impaired voluntary movement [4], [5]. Accurate assessment of spasticity is essential for understanding reflex excitability and guiding effective rehabilitation strategies [3], [6]. However, current clinical tools, including the Modified Ashworth Scale (MAS) and the Tardieu Scale, are examiner-dependent, show limited inter-rater reliability, and lack sensitivity to subtle reflex changes, which restricts their utility in treatment planning [6], [7], [8], [9]. Spasticity involves not only increased reflex response magnitude but also heightened sensitivity to mechanical input, often conceptualized as a reduced reflex threshold [10]. Accordingly, SRTs represent the minimal mechanical input required to elicit a detectable reflex response and serve as quantitative measures of spasticity [11]. SRTs have been estimated using two main approaches: joint rotation–based and tendon tapping–based paradigms [12], [13]. In the joint rotation paradigm, the threshold corresponds to the joint angle at which a reflex response first appears during passive stretch [13]. In the tendon tapping protocol, the deep tendon reflex (DTR) is elicited by a controlled tap, and mechanical input is represented by indentation depth. The threshold corresponds to the minimal indentation depth required to elicit a reflex response [10], [12], [13]. Compared with joint-rotation paradigms that produce global limb stretch and involve multi-joint kinematics and gravity-related torque, tendon tapping provides a focal and mechanically controlled input at the tendon site. Although tendon tapping is routinely used in neurological examinations to qualitatively assess DTRs, its quantitative application for SRTs remains underdeveloped [12].

Beyond clinical grading, quantitative approaches have been developed to assess stretch reflex responses using EMG and force measurements [9], [14]. EMG reflects neural activation, whereas reflex force represents mechanical output at the tendon tap site [5], [15].

Although these approaches quantify neural activation or global mechanical output, they do not directly measure the local structural response of the musculotendon unit. EMG measurements are susceptible to variability in electrode placement, signal noise, skin–electrode impedance, and crosstalk from adjacent muscles. Force measurements reflect global mechanical output and may include contributions from multijoint torques [15]. Consequently, SRTs derived from EMG or force measurements alone do not account for local structural conditions at the site of mechanical input. Indentation depth–based SRTs may not reflect subject-specific tendon compliance, muscle architecture, or overlying soft tissue thickness, which can vary substantially after stroke [16], [17]. A structure-informed measure of reflex response may therefore improve physiological interpretability and cross-subject comparability of SRT estimates.

To address the limited structural interpretability of conventional EMG- and force-based SRT estimates, we propose an ultrasound-derived measure of local tendon deformation during tendon tapping. EMG- and force-based measurements typically require dedicated instrumentation and technical expertise, whereas ultrasound imaging is commonly used in clinical and rehabilitation settings and enables direct visualization of musculoskeletal structures [17].

Whereas tendon indentation depth represents the externally applied mechanical input at the skin surface, T2B displacement reflects the internal structural response of the musculotendon unit. By linking applied mechanical input to ultrasound-quantified tissue deformation [18] and building on prior ultrasound-based studies of muscle architecture in stroke [19], this method establishes a structure-informed framework for SRT estimation across individuals. Previous ultrasound studies have examined structural and mechanical alterations associated with post-stroke spasticity, including reduced fascicle length, altered pennation angles, and increased muscle stiffness [16], [20], [21]. Despite these advances, it remains unclear whether the internal mechanical response of the musculotendon unit during tendon tapping can be used to identify SRTs. We hypothesize that increasing indentation depth initially engages passive tissue compliance and subsequently triggers reflex-mediated contraction, resulting in a measurable transition in T2B deformation that reflects reflex recruitment. In this study, we employ B-mode ultrasound imaging to quantify changes in distal biceps tendon position relative to the bone in stroke survivors. We interpret T2B displacement as a structural index of indentation-induced tissue deformation without assuming direct correspondence to global muscle fascicle length.

This study develops and evaluates an ultrasound-derived method to identify SRTs based on T2B reflex amplitude in individuals with post-stroke spasticity. Specifically, we (i) characterize the relationship between T2B reflex amplitude and indentation depth to identify SRTs; (ii) quantify correlations between T2B reflex amplitude and established force- and rectified integrated EMG (RIEMG)-based reflex measures; (iii) assess the within-session repeatability of T2B-based SRT estimates; and (iv) evaluate whether baseline T2B preload distance is associated with inter-individual variability in SRT. By integrating ultrasound-derived structural information with conventional neuromechanical measures, this method enables structure-informed estimation of SRTs that complements established EMG and force measurements.

## II. Methods

### A. Participants

Ten chronic hemiparetic stroke survivors with clinically identified spasticity (7 males, 3 females; age: 61.9 ± 11.8 years; post-stroke duration: 13.9 ± 9.9 years; Supplementary Table SI) were recruited from the Clinical Neuroscience Research Registry at the Shirley Ryan AbilityLab. Participants met the following inclusion criteria: (i) ≥ 6 months post-stroke with a single unilateral hemispheric lesion; (ii) not enrolled in concurrent upper-extremity studies; and (iii) MAS ≥ 1 on the affected side. The study was approved by the Northwestern University Institutional Review Board (STU00219273). All participants provided written informed consent in accordance with the Declaration of Helsinki.

### B. Experimental Setup and Data Acquisition

#### 1) Integrated Tapping-Sensing Measurement System:

**Fig. 1.**
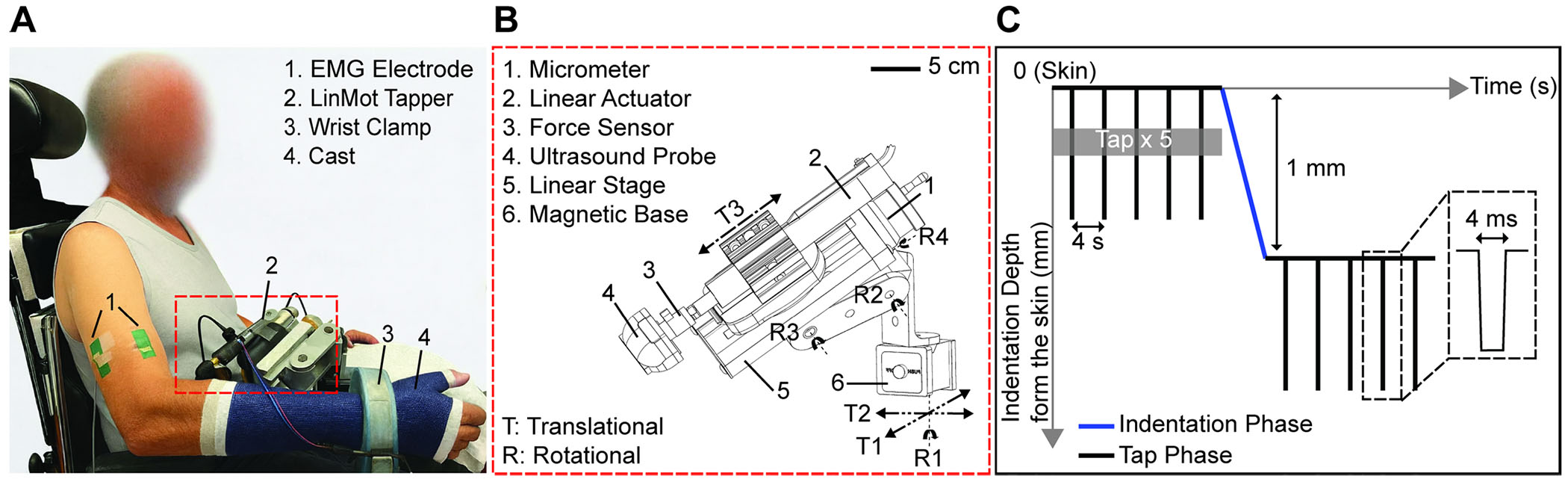
Ultrasound-derived system and experimental protocol. (A) Experimental setup illustrating the LinMot tapper positioned over the distal biceps tendon, an ultrasound probe, and surface EMG sensors over the biceps and triceps brachii. (B) Isometric view of the LinMot tapper, illustrating the linear actuator, a depth-calibration micrometer, a force sensor, and a B-mode ultrasound probe mounted on a linear stage with a magnetic base. The device provides translational (T1–T3) and rotational (R1–R4) degrees of freedom. (C) Schematic illustration of the indentation–tapping protocol applied to the tendon using a LinMot tapper equipped with an ultrasound probe. The actuator followed a predefined sequence that began at the skin surface (indentation depth = 0 mm). Indentation depth increased in 1-mm increments. At each level, the tapper delivered five taps separated by 4-s intervals. Each rectangular tap had a 1-mm amplitude and a 4-ms duration to ensure that the imposed mechanical input was completed before the short-latency reflex response (15–20 ms).

The experimental device integrated a B-mode ultrasound probe at the tapping tip, enabling simultaneous tendon tapping and ultrasound imaging (Fig. 1A, 1B) [12]. A 7.5-MHz ± 35% linear array probe (128 elements) operated at 20 frames/s with an imaging depth of 60 mm (∼0.1 mm/pixel). The probe was part of a custom research ultrasound system developed by the Yongping Zheng group at The Hong Kong Polytechnic University. The probe was placed transversely over the distal biceps tendon to track T2B distance with acoustic coupling gel applied to ensure optimal contact. A custom 3D-printed bracket secured the probe to the LinMot tapper and maintained a stable imaging plane. Imaging parameters were fixed across subjects and trials.

The device also included a high-resolution linear actuator (PS01-23 × 80, LinMot S.A., Spreitenbach, Switzerland) and a custom frame with multiple translational and rotational degrees of freedom that enabled precise spatial positioning (Fig. 1B). One linear axis aligned with the actuator was manually controlled using a micrometer stage (Velmex, Bloomfield, NY, USA). This axis provided 0.1 mm resolution, enabling indentation depth increments of 1 mm. Indentation depth was defined relative to initial skin contact, and subsequent increments were applied from this reference position. Actuator motion was regulated using a proportional–integral–derivative (PID) controller. A single-axis load cell (Model 31, Honeywell Sensotec, Columbus, OH, USA) measuring ±50 N in both compression and tension was positioned behind the tapper head to measure reflex force during each tap. Muscle activity was recorded using single-differential surface EMG sensors (DE- 2.1, Delsys, Natick, MA, USA) connected to a Bagnoli amplifier. Sensors were positioned over the biceps brachii (two channels; medial and lateral heads) and the triceps brachii (one channel). The biceps channel showing the most prominent reflex response across repeated taps was selected for analysis. Skin was cleaned with alcohol prior to electrode placement.

#### 2) Experimental Protocol:

T2B displacement, force, and EMG data were recorded during the same tapping trials (Fig. 1). Participants were seated in an adjustable chair (Biodex Medical Systems, Shirley, NY, USA), with the torso supported to minimize compensatory movements. The forearm was immobilized in a fiberglass cast secured to a custom magnetic base mounted on a steel table (Fig. 1B). Limb posture was standardized at 45° shoulder abduction, 20° shoulder flexion, 120° elbow extension, and a neutral forearm position (Fig. 1A).

The LinMot tapper was positioned perpendicular to the distal biceps tendon, and the zero-depth position was defined at the skin surface. The tapper and ultrasound probe were fixed to maintain consistent targeting across repeated measurements. Each trial consisted of a combined indentation–tapping sequence (Fig. 1C). Starting from the skin surface, the tapper advanced in 1-mm increments. At each depth, five rectangular taps (4 ms duration each) were delivered with 4-second intervals between sequences to allow the musculotendon unit to stabilize. Maximum indentation depth ranged from 15–24 mm, depending on comfort and tissue compliance. Two trials were performed on the affected side with a 5–10 min rest between trials to minimize carryover effects such as fatigue and reflex facilitation.

### C. Data Processing and Analysis

#### 1) Ultrasound-Derived T2B Distance Tracking:

We manually initiated ultrasound recording in parallel with EMG and force acquisition because hardware synchronization and frame-level timestamps were unavailable. Ultrasound frames were aligned to tap events using the fixed frame rate and an estimated start time based on the indentation-phase onset in the EMG and force signals. This alignment procedure introduced a temporal uncertainty of ±1 frame (50 ms). SRT estimation relied on peak T2B amplitude as a function of indentation depth rather than precise onset timing. Therefore, this framelevel uncertainty is unlikely to affect SRT determination.

**Fig. 2.**
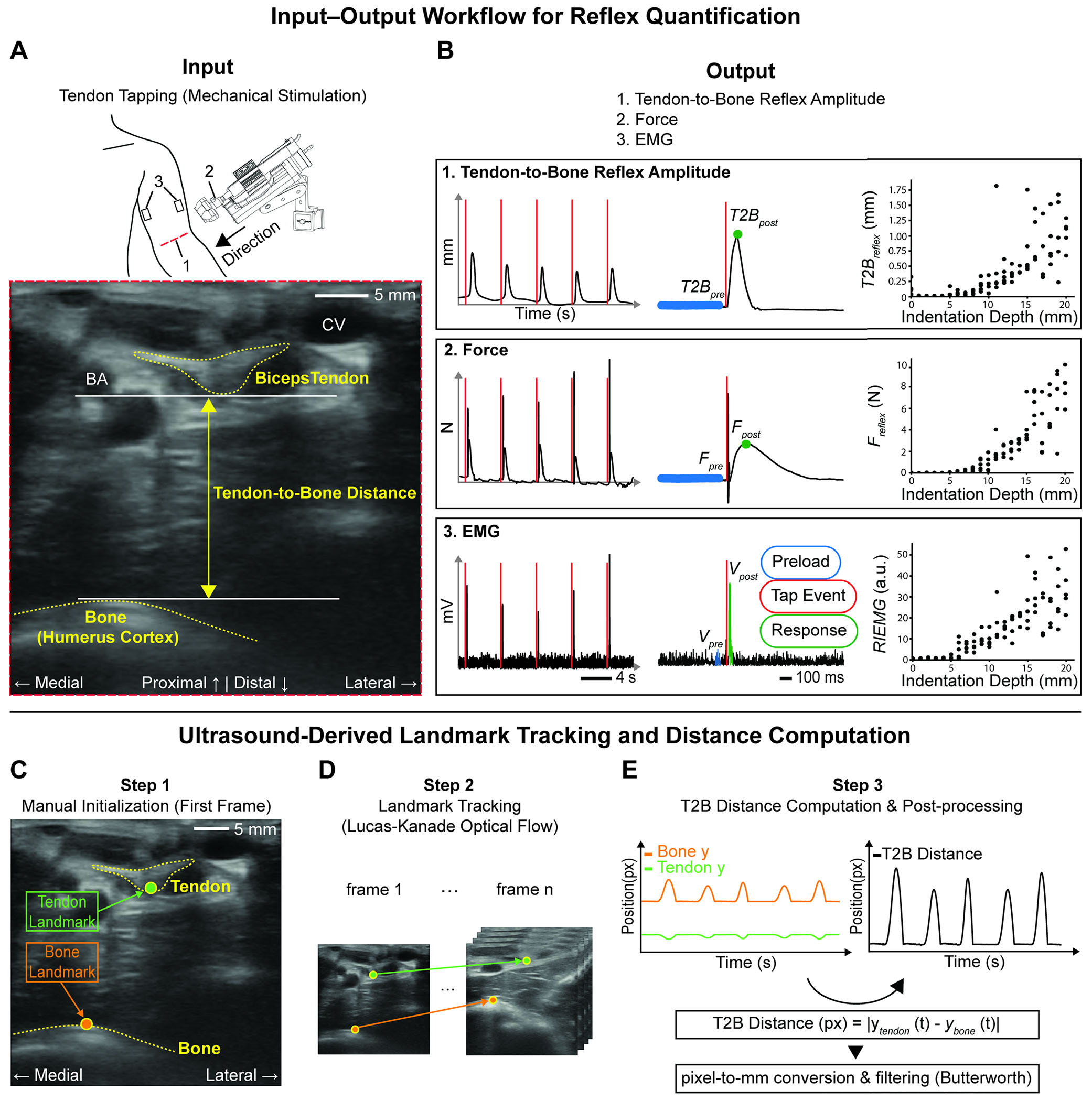
Input–output workflow for reflex quantification and ultrasound-derived T2B distance computation. (A–B) Tendon tapping and multimodal sensing of reflex responses. (A) LinMot tapper, equipped with a B-mode ultrasound probe, delivered controlled taps to the distal biceps tendon using a predefined indentation–tapping protocol. T2B distance was defined as the vertical distance between the distal biceps tendon boundary and the underlying cortical bone surface. (B) Reflex responses were quantified as T2B reflex amplitude, force, and EMG signals computed from modalityspecific pre- and post-tap windows. (C–E) Ultrasound-derived landmark tracking and distance computation. (C) Tendon and bone landmarks are manually initialized on the first ultrasound frame. (D) Landmark positions in subsequent frames are estimated using pyramidal Lucas–Kanade optical flow, with manual correction applied when needed. (E) Frame-wise T2B distance is converted to millimeters and low-pass filtered. Abbreviations: BA, Brachial Artery; CV, Cephalic Vein.

Two anatomical landmarks were manually initialized in the first frame of each trial: one placed on the hyperechoic distal biceps tendon boundary and the other on the humeral cortical surface (Fig. 2A, 2C). All initialization and manual corrections were performed by a single trained rater using a standardized protocol. The landmarks were tracked across all subsequent frames. Inter-frame displacement was estimated using a pyramidal Lucas–Kanade (LK) optical flow [22], [23]. Tracking quality was monitored using predefined drift-detection criteria, including excessive frame-to-frame displacement or LK tracking failure. Drift was flagged when (i) mean landmark displacement >10 pixels; (ii) the displacement of either landmark >1% of frame height; or (iii) tracking failed (status = 0) (Fig. 2D). Manual correction was applied only when flagged frames showed visible deviation from the anatomical boundary. When drift criteria were met, both landmarks were manually realigned to the anatomical boundaries. Tracked and corrected positions were displayed simultaneously to ensure accurate repositioning. Manual correction was limited to localized segments (∼4% of frames) and applied only when tracking produced implausible landmark positions. Otherwise, tracked values were retained.

Tapping stimulation primarily induces vertical motion. Therefore, *T*2*B_raw_*(*t*) was computed as the absolute vertical distance between landmarks and converted to millimeters using system pixel-to-millimeter scaling (Fig. 2E). A second-order Butterworth low-pass filter was applied to suppress tracking noise. The cutoff frequency (4 Hz) was selected based on an amplitude-preservation criterion. Specifically, the lowest common cutoff across subjects was selected to limit changes in T2B reflex amplitude to ≤ 2%. We applied zero-phase filtering using bidirectional filtfilt to avoid phase distortion [24], [25]. The filtered *T*2*B* (*t*) signal was aligned to tap events using the LinMot tapper trigger. For each tap, a 500 ms pre-tap baseline and a 100 ms post-tap response window were defined. T2B reflex amplitude (*T*2*B_reflex_*) was calculated by subtracting the preload T2B distance (*T*2*B_pre_*) from the peak post-tap T2B distance (*T*2*B_post_*).

#### 2) Reflex Force and RIEMG Signal Processing:

For measuring force and EMG signals, we used a Power 1401 mkII system with an ADC 16 expansion module (Cambridge Electronic Design Ltd., Cambridge, UK). The tapping profile consisted of a custom voltage waveform (Fig. 1C) that produced brief rectangular tapping pulses at the actuator tip. This waveform was generated as an analog voltage command through the system’s D/A converter output and transmitted to the LinMot servo drive to control the tip of the LinMot tapper position. Simultaneously, force and EMG signals were digitized at 2 kHz and synchronized using Spike2 software (v8.01, Cambridge Electronic Design Ltd., Cambridge, UK). Participants were instructed to remain fully relaxed. EMG activity was continuously monitored to ensure the absence of voluntary activation throughout the protocol.

Reflex force (*F_reflex_*) and RIEMG were computed to quantify stretch reflex responses. Data processing was performed using custom MATLAB scripts. Second-order zero-phase Butterworth filters were applied to all signals. Force signals were low-pass filtered (cutoff: 450 Hz) to attenuate high-frequency noise, followed by a band-stop filter (0.1–2 Hz) to reduce physiological noise, including respiration and heartbeat interference. *F_reflex_* was calculated by subtracting the preload force (*F_pre_*) from the response force (*F_post_*). *F_pre_* was determined as the mean value over a 500 ms window preceding the tap (blue line in Fig. 2B), while *F_post_* was computed as the mean force over a 100 ms window centered on the peak post-tap response (green line in Fig. 2B). EMG signals were filtered using a band-pass filter (20–450 Hz) to remove motion artifacts and high-frequency noise, and a band-stop filter (60 Hz and its harmonics) to attenuate power line interference. The filtered signals were full-wave rectified and divided into two phases: the preload phase before the tap and the response phase after the tap, as shown in Fig. 2B. The preload phase reflects the resting state of the tendon, whereas the response phase captures the reflex response. RIEMG was calculated using the equation:

(1) $$\text{RIEMG}=\frac{\sum V_{\text{post}}-\sum V_{\text{pre}}}{\sum V_{\text{pre}}},$$

where *V_pre_* and *V_post_* represent the integrated rectified voltage values during the preload and response phase, respectively. RIEMG quantified the normalized change in EMG activity relative to preload. For *V_post_*, the integration window was 20 ms (40–60 ms post-tap). Although stretch reflex responses typically emerge around 20 ms post-tap, we selected 40 ms as the starting point to avoid tap-induced transient artifacts. For *V_pre_*, a 20 ms pre-tap window with minimal standard deviation was selected.

#### 2) SRT Estimation:

We estimated SRT for each modality by fitting a model to the response amplitudes across indentation depths. SRT was defined as the transition point (*x*_1_) of the fitted input–output curve for each modality. Based on the response profile observed in a previous study [18], a piecewise exponential–linear model was fitted to each modality as a function of indentation depth *x*. The exponential–linear model was selected to capture the shallow increase at low indentation depths and the approximately linear increase beyond the reflex threshold. The model was defined as

(2) $$f(x)=\{\begin{array}{cc} a_{1}e^{b_{1}x}+c_{1} & x\leq x_{1}\\ a_{2}x+c_{2} & x>x_{1} \end{array},$$

where *a*_1_, *b*_1_, and *c*_1_ describe the exponential growth in the shallow indentation region, and *a*_2_ denotes the slope of the post-SRT linear response. The intercept *c*_2_ of the linear segment was chosen to ensure value continuity at the transition point,

(3) $$c_{2}=a_{1}e^{b_{1}x_{1}}+c_{1}-a_{2}x_{1},$$

which ensures that *f*(*x*) remained continuous at *x* = *x*_1_. We treated the post-threshold slope *a*_2_ as an independent parameter, allowing for a change in slope at *x*_1_ to capture a transition in the indentation–response relationship. The post-threshold slope *a*_2_ was constrained to be non-negative to reflect the expected monotonic increase of reflex amplitude with increasing indentation depth. The transition point *x*_1_ was constrained to lie within the measured indentation range, excluding a small margin at both ends to avoid boundary solutions.

The same model was applied to all three modalities to ensure that differences in the estimated SRT reflected modality-specific responses rather than model-dependent differences. Preprocessing was performed consistently across modalities, with modality-specific filters applied only when required by the inherent characteristics of each signal. Nonnegativity clipping was applied to T2B and RIEMG amplitudes prior to fitting, as negative values arise from noise or baseline fluctuations and are not physiologically interpretable. Reflex force was not clipped to preserve bidirectional deviations around the baseline. For model fitting, responses recorded at identical indentation depths were aggregated by their mean prior to fitting. The model parameters (*a*_1_, *b*_1_, *c*_1_, *a*_2_, *x*_1_) were estimated using bounded nonlinear least squares with a soft-L1 loss function [26]. To account for potential heteroscedasticity across indentation depths, residuals were weighted by the inverse of the squared bin-wise standard deviation [27]. Binwise standard deviations were floored to a small positive value to prevent excessively large weights in bins with near-zero variance.

### D. Statistical Analysis

Statistical analyses were performed using Python (version 3.11, Python Software Foundation, USA) and MATLAB (R2025b, MathWorks, Natick, MA, USA). Pearson correlation coefficients were used to assess linear associations between T2B reflex amplitude and both reflex force and RIEMG. Correlations were computed separately for each subject and trial across indentation depths. Correlation coefficients were Fisher z-transformed before averaging across trials and subjects and back-transformed for reporting. Given the limited number of planned comparisons and the exploratory nature of the analyses, no multiple-comparison correction was applied. Data were visually inspected for approximate linearity and the absence of extreme outliers. Trial-to-trial variability was evaluated descriptively using mean ± SD and absolute differences because of the small sample size and the focus on within-subject consistency. Agreement between modalityspecific SRT estimates was assessed using Bland–Altman analysis [28], [29]. For each subject, SRT estimates were defined as the average transition depth (*x*_1_) across two trials. Pairwise comparisons were performed between T2B- and force-based SRTs and between T2B- and RIEMG-based SRTs. Mean bias and 95% limits of agreement (LoA, mean ± 1.96 SD) were computed. Within-subject variability was quantified from trial-level SRT estimates obtained from two repeated trials. Between-subject variability was characterized across participants using mean ± SD and coefficients of variation (CV). The relationship between subject-level baseline T2B preload distance and T2B-based SRT was evaluated using both Pearson and Spearman correlation coefficients to assess linear and monotonic associations.

## III. Results

### A. T2B Reflex Amplitude Shows a Threshold Transition With Increasing Indentation Depth

**Fig. 3.**
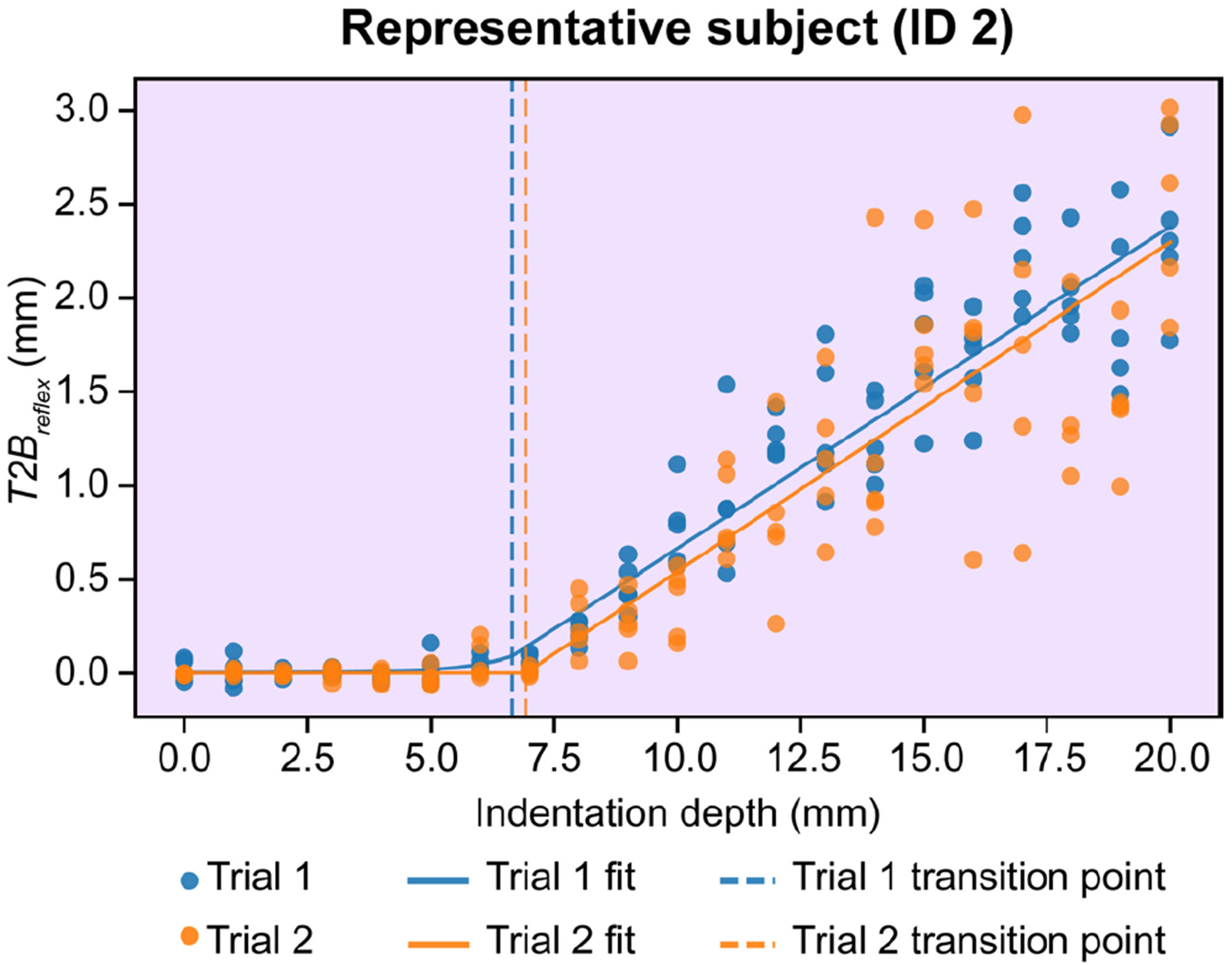
Nonlinear-to-linear transition of T2B reflex amplitude with increasing indentation depth in a representative subject (ID 2). Solid curves indicate piecewise exponential–linear model fits, and vertical dashed lines denote the estimated T2B-based SRT (*x*_1_) for two repeated trials.

**Fig. 4.**
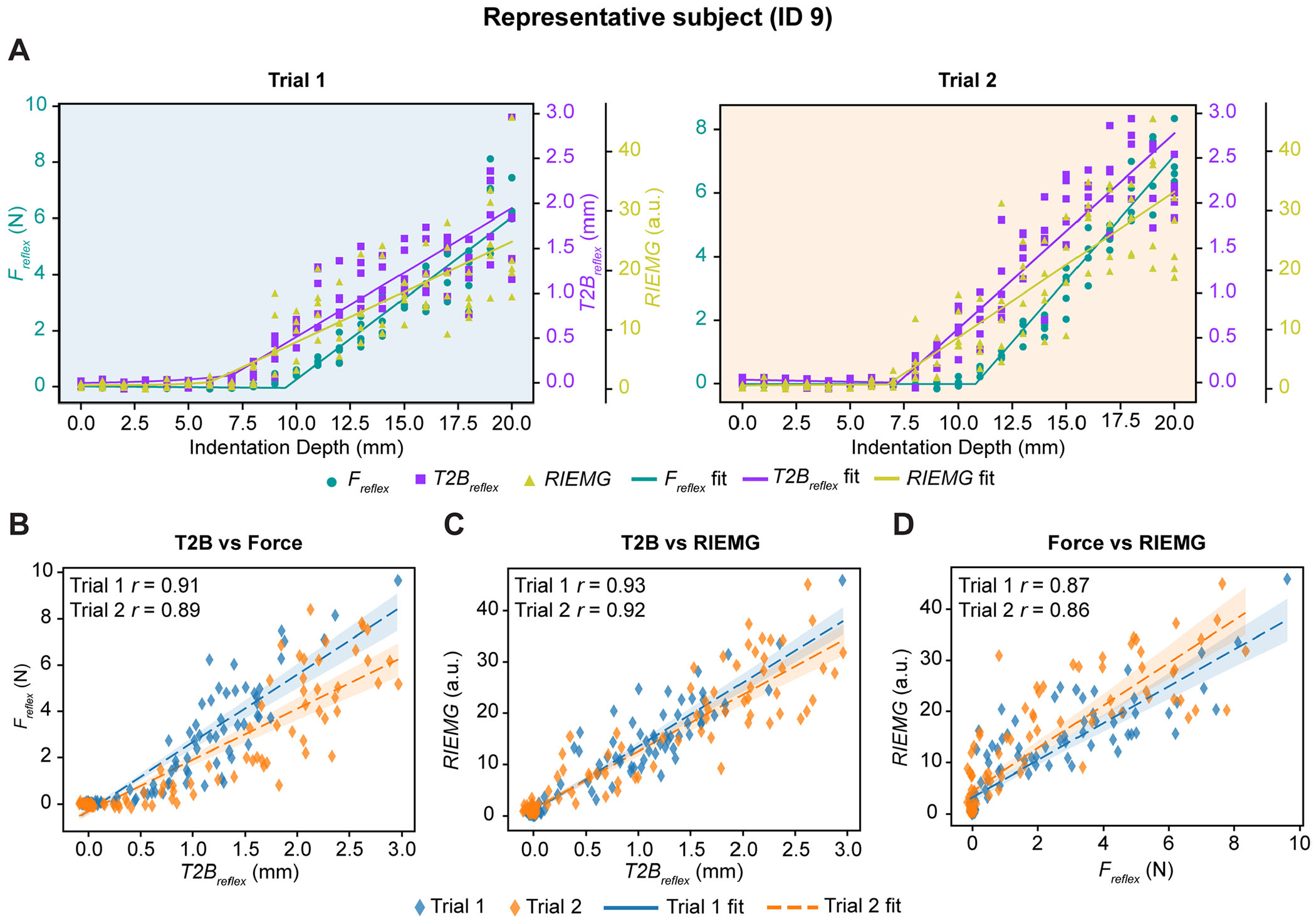
Depth-dependent and within-subject relationships among T2B reflex amplitude, reflex force, and RIEMG in a representative subject (ID 9). (A) Indentation depth–dependent changes in T2B reflex amplitude, reflex force, and RIEMG measured across two repeated trials (Trial 1 and 2). Solid lines indicate piecewise exponential–linear model fits illustrating the nonlinear-to-linear growth pattern of each response. (B–) Within-subject linear correlations between response amplitudes: (B) T2B reflex amplitude vs. reflex force, (C) T2B reflex amplitude vs. RIEMG, and (D) reflex force vs. RIEMG.

In a representative subject (ID 2), T2B reflex amplitude remains minimal at shallow depths and increases sharply beyond a transition point (*x*_1_) (Fig. 3). Force and RIEMG responses show depth-dependent increases consistent with a nonlinear-to-linear transition (Fig. 4A) [30].

### B. Correlations Among T2B Reflex Amplitude, Reflex Force, and RIEMG

Fig. 4 shows representative within-subject relationships for a chronic stroke survivor (ID 9). As indentation depth increases, T2B reflex amplitude and RIEMG exhibit nonlinear-to-linear transitions, whereas reflex force increases more gradually (Fig. 4A). Within this subject, response amplitudes across modalities increase with indentation depth. Strong positive correlations are observed among T2B reflex amplitude, reflex force, and RIEMG (Fig. 4B-D). Across subjects, T2B reflex amplitude is strongly correlated with reflex force (mean *r* = 0.86) and RIEMG (mean *r* = 0.82), comparable to the force–RIEMG correlation (mean *r* = 0.83) (Table I).

### C. Within-Session Repeatability and Agreement of SRT Estimates

Supplementary Table SII summarizes SRT estimates from T2B, force, and RIEMG measurements for each subject. For each modality, SRT estimates from two repeated trials within the same session are reported together with the mean and the absolute trial-to-trial difference (Δ). Across subjects, SRT estimates varied substantially, whereas within-subject differences were smaller.

**Fig. 5.**
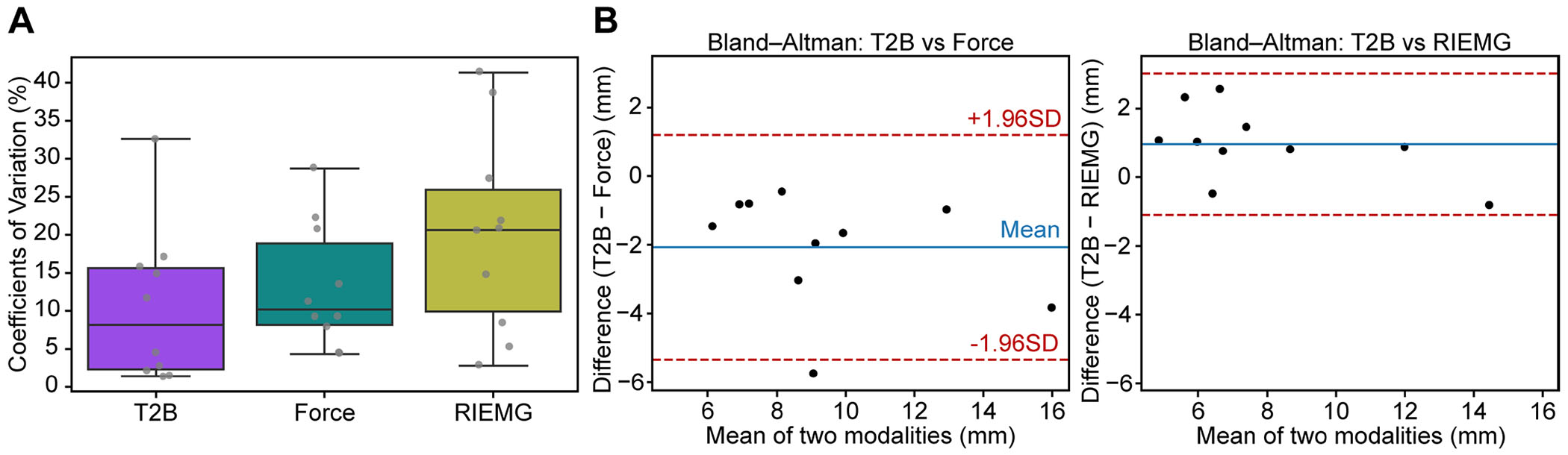
Within-session repeatability and cross-modality agreement of SRT estimates. (A) Box plots summarize the distribution of within-subject coefficients of variation (CV) for T2B-, force-, and EMG-based SRTs across subjects. Each data point represents one subject. T2B- and force-based SRTs exhibit comparable variability, whereas RIEMG-based SRTs show greater dispersion. (B) Bland–Altman plots illustrate agreement between subject-level T2B-based SRTs and force-based (left) and RIEMG-based (right) SRTs. Solid lines indicate the mean difference (bias), and dashed lines indicate the 95% limits of agreement (mean ± 1.96 SD).

Within-session repeatability was quantified using CV computed from the two repeated trials. As shown in Fig. 5A and Supplementary Table SIII, T2B-based SRTs show lower within-subject CV than RIEMG-based SRTs and comparable CV to force-based SRTs. The mean ± SD CV was 10.48 ± 10.06% for T2B-based SRTs, compared with 13.11 ± 8.16% for force-based SRTs and 20.13 ± 13.08% for RIEMG-based SRTs. Bland–Altman analysis shows agreement between T2B-based SRTs and both force- and RIEMG-based SRTs (Fig. 5B). Compared with force-based SRTs, T2B-based SRTs are, on average, 2.1 mm lower, with 95% LoA ranging from −5.34 to 1.20 mm. The mean bias between T2B- and RIEMG-based SRTs is 0.9 mm (95% LoA: −1.13 to 2.99 mm). The mean bias between force- and RIEMG-based SRTs is 3.0 mm (95% LoA: 1.04 to 4.97 mm).

### D. Relationship Between Baseline T2B Preload Distance and SRT

**Fig. 6.**
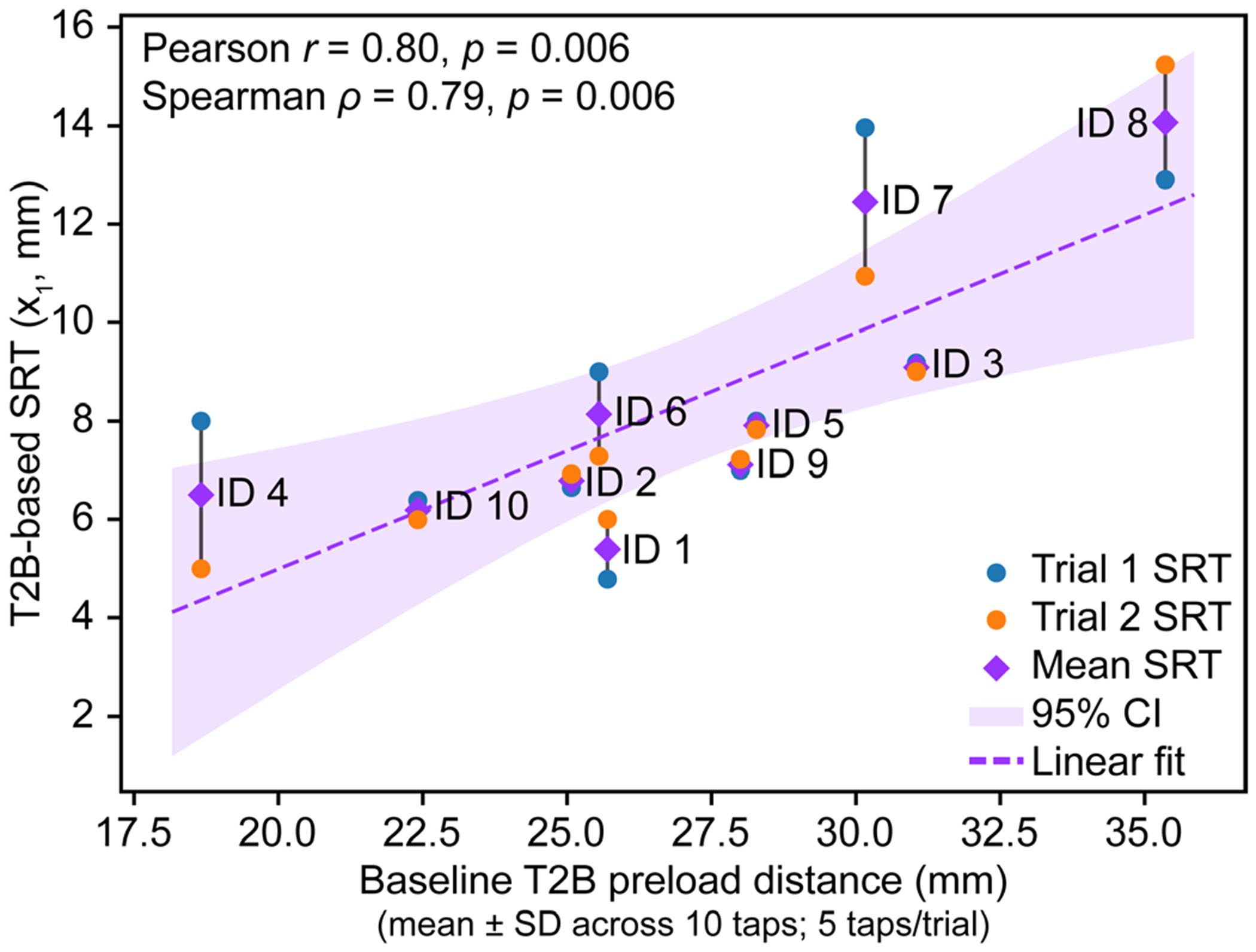
Relationship between baseline T2B preload distance and T2B-based SRT across subjects. Each data point represents one subject (ID 1–10), plotted using the subject-level mean baseline T2B preload distance measured at indentation depth = 0 mm (x-axis) and the corresponding T2B-based SRT (*x*_1_, *mm*) estimated for each subject (y-axis). Horizontal error bars indicate the standard deviation of baseline T2B preload distance across repeated taps, reflecting within-subject measurement variability. The dashed line denotes the linear regression fit, and the shaded region indicates 95% confidence interval.

The relationship between baseline T2B preload distance and T2B-based SRT was examined. Baseline T2B preload distance was quantified at the skin surface (indentation depth = 0 mm) and averaged across repeated taps from two trials (10 taps per subject; 5 taps per trial). As shown in Fig. 6, baseline T2B preload distance varies substantially across subjects. Across subjects, baseline T2B preload distance is significantly correlated with T2B-based SRT (*r* = 0.80, *p* = 0.006). This correlation remains significant using Spearman rank correlation (*ρ* = 0.79, *p* = 0.006). Similar correlation magnitudes are observed when the two trials are analyzed separately (Supplementary Fig. S2). Trial-level baseline T2B preload distances and corresponding SRT estimates for each subject are summarized in Supplementary Table SIV.

## IV. Discussion

This study introduces an ultrasound-derived method for estimating SRTs based on T2B reflex amplitude during controlled tendon tapping in chronic post-stroke spasticity. Across subjects, T2B reflex amplitude exhibits a consistent transition with increasing indentation depth and shows strong correlations with reflex force and RIEMG, similar to established measures of stretch reflex responses. T2B-based SRTs show within-session repeatability comparable to forcebased SRTs and lower variability than RIEMG-based SRTs. Additionally, baseline T2B preload distance varies across individuals and is associated with T2B-based SRT, suggesting that subject-specific structural configuration may contribute to inter-individual differences in reflex sensitivity.

### A. Physiological Basis of Ultrasound-Derived T2B Reflex Measures

T2B distance captures localized tendon–bone separation at the site of tendon tapping [18], [31], [32]. Tendon tapping activates muscle spindle receptors, which transmit neural signals to the spinal cord and result in motor unit recruitment and subsequent force development within the musculotendon unit. Different measurement modalities capture distinct stages of this process [33]. Surface EMG reflects neural output following spindle activation and synaptic transmission, whereas reflex force reflects the mechanical response transmitted to the probe–tissue interface [34].

In contrast to EMG or force, T2B displacement reflects local tissue motion relative to a bone reference. Although T2B displacement and reflex force are elicited by the same controlled tap, they represent different mechanical levels of the response. Reflex force is measured by the load cell mounted behind the probe and reflects the net reaction force transmitted through the probe–tissue interface, whereas T2B displacement reflects local internal tendon motion relative to the humeral cortex. Indentation depth establishes the initial mechanical loading condition of the musculotendon unit, whereas T2B reflex amplitude quantifies the tap-induced deformation relative to the immediately pre-tap baseline. To minimize contributions from slow drift or tissue creep, T2B reflex amplitude is defined as the post-tap peak change relative to the immediately pre-tap baseline within a short post-tap window.

T2B displacement, therefore, captures local changes in tendon–bone separation in response to indentation, including contributions from both passive tissue deformation and reflex-mediated contraction. The observed nonlinear-to-linear transition in T2B reflex amplitude occurs in parallel with concurrent EMG and force responses (Fig. 4B-D), consistent with its association with reflex recruitment rather than passive mechanical effects alone. Accordingly, the SRT estimate proposed here is derived from systematic changes in reflex amplitude across indentation depth rather than from millisecond-scale latency measurements of individual reflex events. Although T2B displacement does not directly estimate muscle fascicle length, it reflects relative changes in tendon–bone separation under a fixed joint configuration. Under standardized posture, these changes provide a structurally consistent index of indentation-induced deformation rather than an absolute measure of muscle length.

### B. Comparison With Conventional Reflex/Spasticity Assessment Methods

The most widely accepted clinical scale for spasticity assessment is the MAS [3]. All subjects in this study had MAS = 1. Despite identical MAS grades, T2B-based SRTs span a wide range (5–14 mm) and are accompanied by intersubject variability in baseline T2B preload distance (Fig. 6 and Supplementary Table SIV). Previous studies report that the MAS provides a coarse ordinal rating scale and may confound neural reflex activation and passive mechanical tissue properties, limiting its ability to isolate reflex excitability across subjects [3], [35], [36], [37]. However, the observed variability may also reflect differences in the level of measurement. Clinical scales such as the MAS and Tardieu Scale assess global responses during passive joint rotation, whereas the proposed T2B-based approach captures local musculotendon responses.

T2B-based measures here are compared with established modalities, including EMG and reflex force. EMG primarily reflects neural activation, whereas reflex force incorporates both active contraction and passive mechanical contributions [4], [34]. To examine the relationship between T2B and force, T2B reflex amplitude was modeled as a function of reflex force and indentation depth. Force and indentation depth explained a substantial portion of T2B variance (*R*^2^ = 0.69), indicating substantial shared mechanical responses between the two measurements. In an exploratory pooled residual analysis, residual T2B showed a weak association with baseline T2B preload distance (*r* = −0.13, *p* = 0.0065). Given the modest effect size, repeated-measures design, and limited number of subjects, this finding should be interpreted cautiously as exploratory support for a possible baseline-dependent structural component of T2B variability.

The greater agreement observed between T2B- and RIEMG-based SRTs compared with force-based SRTs may reflect differences in the physiological and mechanical components captured by each modality. RIEMG primarily reflects neural activation associated with the stretch reflex, whereas force measurements include additional passive and geometric contributions. T2B displacement captures localized tendon deformation associated with reflex-mediated loading. This characteristic may explain the closer agreement between T2B- and RIEMG-based SRTs relative to force-based SRTs.

Consistent with this interpretation, Bland–Altman analysis (Fig. 5B) shows closer agreement between T2B- and RIEMG-based SRTs, whereas force- and RIEMG-based SRTs exhibit the largest bias. Although the MAS remains a practical and efficient clinical tool, the present results indicate variability in T2B-based SRTs within a single MAS grade. These findings suggest that quantitative SRT measures may capture variability not represented by ordinal clinical grading. Therefore, the observed SRT variability should be interpreted with caution because it cannot be clearly linked to clinical severity in the present cohort and may reflect subject-specific anatomical or structural differences.

### C. Within-Session Repeatability and Practical Advantages of the Proposed Method

The proposed T2B-based method demonstrates within-session repeatability under fixed measurement conditions, as reflected by the variability of T2B-, force-, and RIEMG-based SRT estimates (Supplementary Table SII). Across subjects, T2B-based SRTs exhibit low within-subject variability, with CV values comparable to those of force-based SRTs and generally lower than those of RIEMG-based SRTs. RIEMG-based SRTs show higher within-session variability, consistent with the known sensitivity of EMG to electrode placement, background muscle activity, skin–electrode impedance, and crosstalk [34], [38].

In addition to within-session repeatability, the T2B-based method offers practical advantages for quantitative reflex assessment through reduced setup complexity and improved accessibility compared with conventional EMG- and force-based measurements. Unlike surface EMG recordings, ultrasound-derived T2B measurements are less susceptible to electrical noise, grounding requirements, and variations in skin–electrode impedance [34]. External force transducers are not required for SRT estimation using this method. Because T2B displacement is derived from anatomical tracking within a defined imaging plane, the method reduces reliance on repeated surface electrode placement. Moreover, ultrasound imaging is widely used in clinical settings and does not require specialized neuromuscular signal acquisition, such as surface EMG or force transduction. In contrast, surface EMG and force measurements typically require dedicated instrumentation and technical expertise for reliable acquisition.

### D. Anatomical Interpretation of Baseline T2B Preload Distance

Baseline T2B preload distance can be interpreted as a primarily passive structural parameter reflecting the resting geometry of the musculotendon unit. Inter-individual differences in baseline T2B preload distance likely arise from variability in muscle and limb bulk, tendon slack, resting tension, passive muscle stiffness, and connective tissue compliance [39], [40]. Baseline T2B preload distance is defined at indentation depth = 0 mm under probe contact prior to each tap. At this level, no consistent reflex-related EMG or force signals are observed, supporting the interpretation that the baseline primarily reflects passive mechanical configuration rather than reflex-induced muscle activation.

Potential measurement-related confounders are considered under the standardized experimental conditions. All measurements are performed in a standardized posture to maintain consistent joint configuration across subjects. Although subcutaneous tissue thickness and probe alignment can influence ultrasound-derived measurements, the present analysis focuses on relative tendon–bone separation within each subject, which reduces sensitivity to absolute depth offsets. Moreover, baseline T2B preload distance shows low variability across repeated taps within each trial and, in most subjects, remains comparable across trials (Supplementary Table SIV). This low within-subject variability suggests that superficial tissue effects or measurement artifacts are unlikely to fully account for the observed inter-subject differences in baseline T2B preload distance, although minor contributions cannot be excluded.

### E. Relationship Between Baseline T2B Preload Distance and SRT

Across subjects, baseline T2B preload distance is positively correlated with T2B-based SRT (Fig. 6), such that greater baseline T2B preload distance is associated with higher SRT estimates. The T2B-based SRT is defined relative to the baseline tendon–bone configuration. Accordingly, baseline T2B preload distance can be interpreted as a structural initial condition, whereas SRT reflects a functional outcome of the neuromechanical system. Consistent with this interpretation, the relationship between baseline T2B preload distance and SRT remains similar when analyzed using either trial-level values or subject-level averages (Fig. 6, Supplementary Fig. S2).

However, some individuals exhibit similar baseline T2B preload distances yet demonstrate different SRTs (Fig. 6). This observation indicates that baseline T2B preload distance alone does not fully explain reflex sensitivity. These findings suggest that SRT may depend on interactions between structural baseline conditions and neural or mechanical system properties. Differences in passive tissue stiffness, tendon compliance, neuromuscular excitability, spinal reflex gain, or musculotendon force transmission may influence how local tendon deformation modulates reflex activation during tendon tapping [37], [40], [41], [42]. Neurophysiological mechanisms are not directly assessed in this study but may contribute to inter-individual differences in SRT and warrant further investigation.

Trial-to-trial changes in SRT within the same individual are accompanied by corresponding differences in baseline T2B preload distance (Supplementary Fig. S2; Table SIV). Although these between-trial shifts were modest in some subjects, they occur in the same direction for both baseline T2B preload distance and SRT. These findings suggest that within-session trial-to-trial variability in SRT may reflect differences in the initial mechanical state of the musculotendon unit rather than being solely attributable to measurement noise. Although the tapper is position-controlled, musculotendon compliance implies that identical actuator displacement may not produce identical internal loading. Unlike actuator-defined indentation depth, which reflects externally imposed displacement, T2B displacement provides an internal structural reference relative to the applied mechanical input. This distinction provides additional physiological context for interpreting SRT estimates.

### F. Limitations and Future Directions

First, the sample size is limited, and all subjects have chronic post-stroke spasticity. Future studies with larger cohorts and additional populations, including neurologically intact individuals and patients with other neurological conditions, will be needed to establish broader generalizability.

Second, the experimental protocol focuses on a single muscle group. Stretch reflex behavior and musculotendon structural properties can vary across muscles with different anatomical structures and functional roles. Extending the proposed T2B-based framework to other muscles and joints will therefore be important for broader clinical applicability.

Third, because the present cohort includes individuals with identical MAS grades (MAS = 1), the observed inter-subject variability reflects differences within a narrow clinical severity range. Future studies that include a broader range of spasticity severity will be needed to examine the relationship between T2B-based measures and clinical grading scales.

Fourth, ultrasound image acquisition is not hardware-synchronized with EMG and force recordings, which limits the precision of frame-level timing of reflex onset. However, the primary outcome measures derive from systematic changes in response magnitude across indentation depth. Future implementations incorporating hardware synchronization or higher-frame-rate ultrasound could enable complementary timing-based analyses.

Fifth, the optical-flow-based tracking used to estimate T2B distance can be sensitive to local speckle variation and image quality. Tracking quality is verified for each trial, and manual corrections are applied only when necessary. These corrections are rare and do not appear to affect SRT estimation. Future work may further improve robustness through enhanced tracking algorithms and automated quality-control procedures.

Sixth, although within-session repeatability is demonstrated under standardized posture, cross-session reproducibility following probe or LinMot tapper repositioning or changes in joint configuration is not directly evaluated. Because T2B-based SRT is defined relative to baseline tendon–bone configuration, variations in limb position or probe alignment may systematically shift threshold estimates. Such shifts reflect changes in the initial mechanical configuration of the musculotendon unit rather than purely geometric measurement error. Thus, the reported within-session variability reflects repeatability under controlled conditions and may not fully represent variability in repeated-measurement settings. Future studies should quantify the sensitivity of SRT to controlled variations in joint posture and device repositioning and evaluate test–retest reproducibility over longer time intervals.

Seventh, although T2B displacement provides a localized structural measure, further work is needed to determine optimal normalization strategies across individuals. Normalization approaches incorporating baseline T2B preload distance and other ultrasound-derived architectural parameters may improve cross-subject comparability. In addition, the relationship between T2B displacement and underlying musculotendon length changes remains to be clarified. Integrating ultrasound-based measurements of muscle architecture may help determine whether T2B displacement reflects localized muscle length changes and how such measures could support physiologically informed normalization across individuals.

Finally, while the current framework prioritizes interpretability, further optimization of the experimental and analysis pipeline, such as automated feature extraction and SRT estimation from T2B distance signals, could reduce manual processing. Future work integrating T2B-based measures with assessments of passive tissue mechanics and neuromuscular excitability may help distinguish structural and neural contributions to SRT variability.

## V. Conclusion

Ultrasound-derived T2B displacement enables quantification of subject-specific SRTs during controlled tendon tapping in post-stroke spasticity. By linking T2B displacement to stretch reflex behavior, the proposed method provides a mechanistically interpretable complement to conventional EMG- and force-based assessments. These findings support a structurally grounded approach to SRT estimation that integrates ultrasound-quantified tissue deformation with conventional threshold identification, providing a complementary tool for quantitative spasticity assessment.

## Supplementary Material

supp1-3705277

## Figures and Tables

**TABLE I:** Subject-Level Correlations Between T2B Reflex Amplitude, Reflex Force, and RIEMG

ID	T2B - Force Pearson r		T2B - RIEMG Pearson r		Force - RIEMG Pearson r	

	Trial 1	Trial 2	Trial 1	Trial 2	Trial 1	Trial 2
1	0.87	0.84	0.87	0.77	0.76	0.80
2	0.93	0.95	0.83	0.92	0.79	0.89
3	0.74	0.82	0.73	0.79	0.90	0.91
4	0.74	0.89	0.73	0.78	0.89	0.77
5	0.94	0.93	0.91	0.88	0.93	0.93
6	0.85	0.87	0.57	0.73	0.43	0.58
7	0.91	0.91	0.92	0.88	0.92	0.82
8	0.69	0.75	0.75	0.60	0.72	0.66
9	0.91	0.89	0.93	0.92	0.87	0.86
10	0.64	0.39	0.57	0.70	0.83	0.73
Mean *r*	0.86		0.82		0.83	

Correlations were computed separately for each trial across indentation depths within each subject. Group-level mean *r* values across subjects were obtained using Fisher z-transformation and back-transformation (n = 10).
