# Supplementary material for: Ultrasound-Derived Stretch Reflex Threshold Estimation Using Tendon-to-Bone Distance During Tendon Tapping in Post-Stroke Spasticity
Source: IEEE Trans Neural Syst Rehabil Eng. Author manuscript; Available in PMC 2026 Jul 25. (PMC13401269; doi:10.1109/TNSRE.2026.3705277)
Supplement: supp1-3705277 [file NIHMS2193247-supplement-supp1-3705277.pdf]

Supplementary Material

Ultrasound-Derived Stretch Reflex Threshold Estimation Using Tendon-to-Bone Distance During Tendon Tapping in Post-Stroke Spasticity

Seongyeon Yang, Matthieu K. Chardon, Vaheh Nazari, Zhen Song, Yongping Zheng, Sungjin Bae, and William Z. Rymer, *Life Member, IEEE*

Contents

- **Supplementary Figures**

Fig. S1 Transverse ultrasound anatomy of the distal biceps tendon
Fig. S2 Relationship between baseline T2B preload distance and T2B-based SRT

- **Supplementary Tables**

Table SI Participant demographics and clinical characteristics
Table SII Subject-specific SRT estimates derived from T2B, force, and RIEMG
Table SIII Trial-to-Trial variability (CV) of T2B-, Force-, and RIEMG-based SRTs
Table SIV Trial-level baseline T2B preload distances and corresponding T2B-based SRT estimates

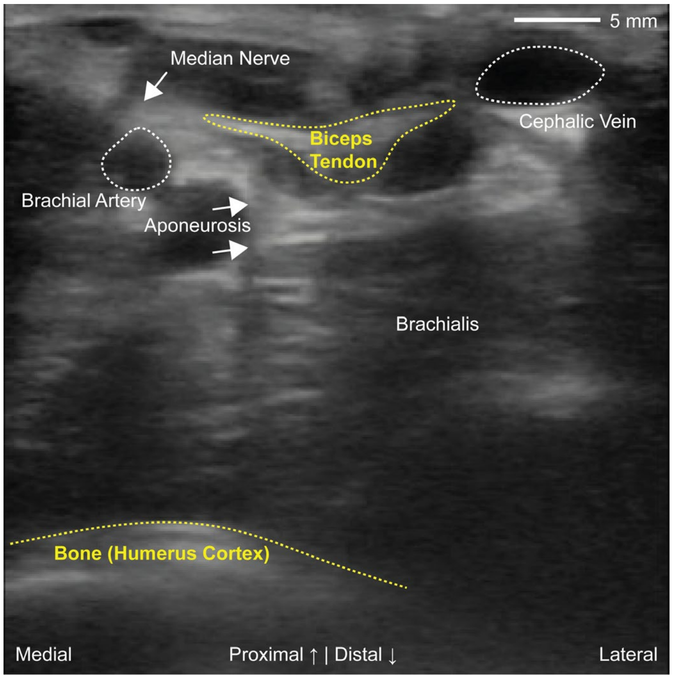

Fig. S1. Transverse ultrasound anatomy of the distal biceps tendon. A representative B-mode ultrasound image illustrates the distal biceps tendon (yellow dotted outline). The brachial artery is located medially adjacent to the median nerve, whereas the cephalic vein is visualized laterally to the tendon. The bicipital aponeurosis appears as a thin hyperechoic band deep to the subcutaneous tissue. The brachialis muscle occupies the deep compartment beneath the tendon, and the humeral cortex is visible as a continuous hyperechoic line representing the bone surface. Axes indicating medial–lateral and proximal–distal orientations are provided for anatomical reference.

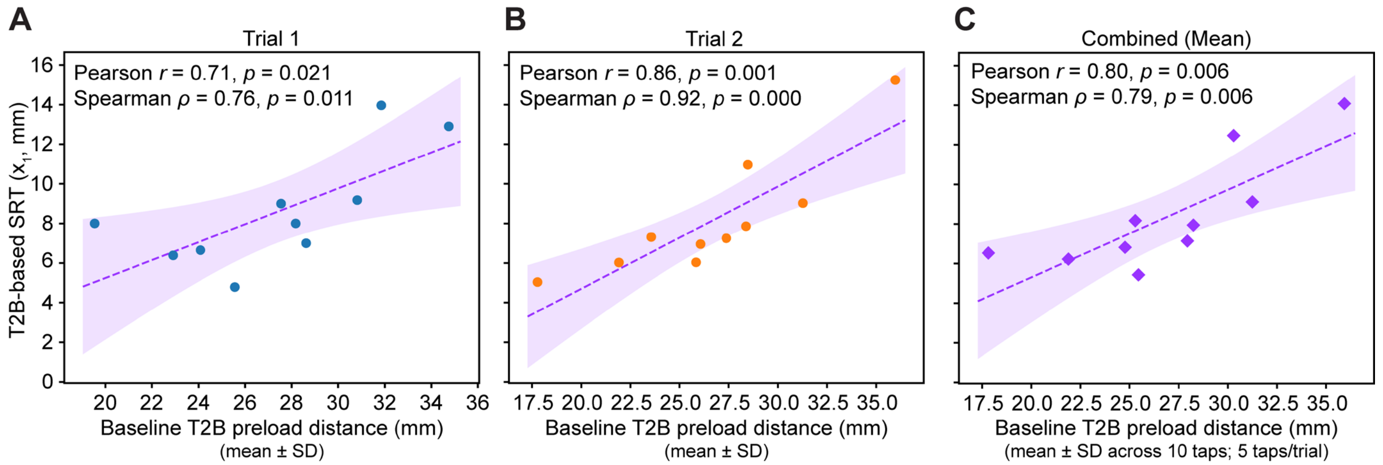

Fig. S2. Relationship between baseline T2B preload distance and T2B-based SRT. Scatter plots illustrate the correlation between baseline T2B preload distance and T2B-based SRT for (A) Trial 1, (B) Trial 2, and (C) subject-level mean values across trials. Dashed lines indicate linear regression fits with shaded regions representing 95% confidence intervals. Pearson and Spearman correlation coefficients with corresponding p-values are shown in each panel.

TABLE SI

PARTICIPANT DEMOGRAPHICS AND CLINICAL CHARACTERISTICS

ID	Sex	Age (years)	Dominant Side	Paretic Side	Duration of Stroke (years)	MAS biceps brachii
1	M	74	Right	Left	15	1
2	M	59	Left	Right	7	1
3	M	74	Right	Left	8	1
4	F	74	Left	Right	40	1
5	M	70	Right	Left	13	1
6	M	56	Right	Left	16	1
7	F	70	Right	Left	14	1
8	M	45	Left	Right	12	1
9	M	44	Right	Left	4	1
10	F	53	Right	Left	10	1

TABLE SII

SUBJECT-SPECIFIC SRT ESTIMATES DERIVED FROM T2B, FORCE, AND RIEMG

ID	T2B				Force				RIEMG			
	Trial 1	Trial 2	Mean	Δ	Trial 1	Trial 2	Mean	Δ	Trial 1	Trial 2	Mean	Δ
1	4.79	6.00	5.40	1.21	6.41	7.30	6.86	0.89	5.00	3.72	4.36	1.28
2	6.65	6.92	6.79	0.27	6.48	8.70	7.59	2.22	3.18	5.81	4.50	2.63
3	9.18	9.00	9.09	0.18	11.08	10.42	10.75	0.66	8.00	8.61	8.31	0.61
4	8.00	5.00	6.50	3.00	8.80	5.83	7.32	2.97	7.00	4.00	5.50	3.00
5	8.00	7.83	7.92	0.17	7.57	9.16	8.37	1.59	4.56	6.22	5.39	1.66
6	9.00	7.28	8.14	1.72	10.89	9.30	10.10	1.59	5.41	8.00	6.71	2.59
7	13.96	10.94	12.45	3.02	15.52	11.31	13.42	4.21	13.28	9.92	11.60	3.36
8	12.90	15.24	14.07	2.34	17.34	18.46	17.90	1.12	14.61	15.20	14.91	0.59
9	7.00	7.22	7.11	0.22	9.48	10.79	10.14	1.31	6.00	6.75	6.38	0.75
10	6.39	5.99	6.19	0.40	11.27	12.59	11.93	1.32	7.39	6.00	6.70	1.39

SRT values are reported for two repeated trials within the same session. Mean values represent the average across trials, and Δ denotes the absolute difference between Trial 1 and Trial 2.

TABLE SIII

TRIAL-TO-TRIAL VARIABILITY (CV) OF T2B-, FORCE-, AND EMG-BASED SRTS

ID	T2B CV (%)	Force CV (%)	RIEMG CV (%)
1	15.86	9.18	20.76
2	2.81	20.68	41.37
3	1.40	4.34	5.19
4	32.64	28.71	38.57
5	1.52	13.44	21.78
6	14.94	11.14	27.31
7	17.15	22.19	20.48
8	11.76	4.42	2.80
9	2.19	9.14	8.32
10	4.57	7.82	14.68
Mean ± SD	10.48 ± 10.06	13.11 ± 8.16	20.13 ± 13.08

Coefficients of variation (CV, %) were calculated within each subject using SRT estimates from the two repeated trials. SRT estimates correspond to the transition point (x_1) identified from the piecewise exponential–linear model fits for T2B reflex amplitude, reflex force, and RIEMG. Group-level values are reported as mean ± SD across subjects (n = 10).

TABLE SIV

TRIAL-LEVEL BASELINE T2B PRELOAD DISTANCES AND CORRESPONDING T2B-BASED SRT ESTIMATES

ID	Baseline T2B Preload Distance (mm)												SRT (mm)	
	Trial 1						Trial 2						Trial 1	Trial 2
	Tap 1	Tap 2	Tap 3	Tap 4	Tap 5	Mean	Tap 1	Tap 2	Tap 3	Tap 4	Tap 5	Mean		
1	25.61	25.62	25.54	25.48	25.55	25.56	25.70	25.85	25.87	25.81	25.97	25.84	4.79	6
2	23.89	23.86	23.90	24.37	24.40	24.08	26.03	26.03	26.02	26.04	26.20	26.06	6.65	6.92
3	30.76	30.81	30.87	30.89	30.75	30.82	31.12	31.15	31.35	31.32	31.44	31.28	9.18	9
4	19.58	19.54	19.52	19.53	19.53	19.54	17.80	17.76	17.77	17.78	17.80	17.78	8	5
5	27.89	28.25	28.20	28.25	28.28	28.17	28.37	28.37	28.39	28.39	28.39	28.38	8	7.83
6	27.83	27.65	27.41	27.34	27.51	27.55	23.57	23.53	23.55	23.56	23.56	23.55	9	7.28
7	31.73	31.73	31.95	31.95	31.87	31.84	28.49	28.47	28.47	28.46	28.47	28.47	13.96	10.94
8	34.76	34.69	34.82	34.72	34.73	34.74	35.95	35.97	35.96	36.01	35.96	35.97	12.9	15.24
9	28.68	28.62	28.57	28.55	28.67	28.62	27.30	27.36	27.38	27.41	27.45	27.38	7	7.22
10	22.79	22.96	22.85	22.97	22.98	22.91	21.81	21.94	21.92	21.95	21.98	21.92	6.39	5.99

Baseline T2B preload distance was measured at indentation depth = 0 mm (skin surface) for each tap and averaged across five taps within each trial.
